# Selective Cardiomyocyte Oxidative Stress Leads to Bystander Senescence of Cardiac Stromal Cells

**DOI:** 10.3390/ijms22052245

**Published:** 2021-02-24

**Authors:** Hélène Martini, Lise Lefevre, Sylvain Sayir, Romain Itier, Damien Maggiorani, Marianne Dutaur, Dimitri J. Marsal, Jérôme Roncalli, Nathalie Pizzinat, Daniel Cussac, Angelo Parini, Jeanne Mialet-Perez, Victorine Douin-Echinard

**Affiliations:** 1 Institute of Metabolic and Cardiovascular Diseases (I2MC), Inserm UMR 1048, University of Toulouse, 31400 Toulouse, France; helene.martini@hotmail.com (H.M.); lise.lefevre@univ-tlse3.fr (L.L.); damien.maggiorani@gmail.com (D.M.); marianne.dutaur@inserm.fr (M.D.); dimitri.marsal@inserm.fr (D.J.M.); roncalli.j@chu-toulouse.fr (J.R.); nathalie.pizzinat@inserm.fr (N.P.); daniel.cussac@inserm.fr (D.C.); angelo.parini@inserm.fr (A.P.); 2 Institute Cardiomet, FHU IMPACT, University Hospital of Toulouse, 31400 Toulouse, France; sayir.sylvain@gmail.com (S.S.); itier.r@chu-toulouse.fr (R.I.)

**Keywords:** stress-induced premature senescence, oxidative stress, monoamine oxidase A, SASP, cardiac mesenchymal stromal cells, CCR2^+^ Macrophages

## Abstract

Accumulation of senescent cells in tissues during normal or accelerated aging has been shown to be detrimental and to favor the outcomes of age-related diseases such as heart failure (HF). We have previously shown that oxidative stress dependent on monoamine oxidase A (MAOA) activity in cardiomyocytes promotes mitochondrial damage, the formation of telomere-associated foci, senescence markers, and triggers systolic cardiac dysfunction in a model of transgenic mice overexpressing MAOA in cardiomyocytes (Tg MAOA). However, the impact of cardiomyocyte oxidative stress on the cardiac microenvironment in vivo is still unclear. Our results showed that systolic cardiac dysfunction in Tg MAOA mice was strongly correlated with oxidative stress induced premature senescence of cardiac stromal cells favoring the recruitment of CCR2^+^ monocytes and the installation of cardiac inflammation. Understanding the interplay between oxidative stress induced premature senescence and accelerated cardiac dysfunction will help to define new molecular pathways at the crossroad between cardiac dysfunction and accelerated aging, which could contribute to the increased susceptibility of the elderly to HF.

## 1. Introduction

Advanced age remains a strong predictor for poor outcomes in patients with chronic or acute heart failure (HF). Age-related changes in cardiac structure and longer exposure to risk factors may render the elderly more prone to develop cardiovascular diseases. Among these factors, the accumulation of reactive oxygen species (ROS) which mainly originate from mitochondria generates oxidative stress, one mechanism known to promote both cardiac dysfunction and stress-induced premature senescence (SIPS) [1,2]. The central role of mitochondrial ROS in cardiac aging was demonstrated in mice overexpressing the antioxidant enzyme catalase targeted to mitochondria, which displayed preserved cardiac performance until 24 months [3]. In the heart, the aging process is to a large extent related to macromolecular damage by ROS, mostly affecting long-lived postmitotic cells such as cardiomyocytes. ROS can directly oxidize proteins involved in contractile activity [4] or interfere with quality control mechanisms such as proteostasis and autophagy, promoting senescence and/or apoptosis [5]. Emerging evidence further suggests that a redox crosstalk may occur between cardiomyocytes and stromal cells during aging through direct or indirect mechanisms [6]. At present, the sources of mitochondrial ROS responsible for age-related cardiac dysfunction are still under investigation.

Recently, we and others have identified the mitochondrial enzyme monoamine oxidase-A (MAOA) as a major source of hydrogen peroxide (H_2_O_2_) in the heart during the metabolism of noradrenaline and serotonin and demonstrated their role in the onset and progression of cardiac injury [7,8,9]. During cardiac aging, increased sympathetic activity contributes to the enhanced release of noradrenaline in the heart. In addition, MAO-A expression in the heart and its ability to produce ROS increase with age [10] and its overexpression in cardiomyocytes drives systolic dysfunction in a transgenic mouse model (Tg MAOA) [9]. We have previously demonstrated that oxidative stress dependent on MAOA activity promotes the formation of telomere-associated foci in cardiomyocytes and induces cardiomyocyte senescence through ROS production, mitochondrial dysfunction, activation of the DDR-p53 pathway, and inhibition of mitophagy [11,12]. We hypothesized that the oxidative stress generated by cardiomyocytes overexpressing MAOA could have an additional deleterious impact on the cardiac microenvironment favoring stress-induced premature senescence of stromal cells. 

With aging, the accumulation of senescent cells in the tissues and acquisition of senescence-associated secretory phenotype (SASP) are known to participate in the loss of tissue homeostasis and to the increased incidence of age-related pathologies such as HF. The SASP is characterized by transcriptomic expression of various secreted factors such as chemokines, cytokines, proteases and growth factors which have autocrine and paracrine effects. SASP is highly heterogeneous and cell-type-specific with dynamic changes occurring over time [13]. SASP related factors such as pro-inflammatory cytokines play a deleterious role in cardiovascular diseases, in particular in the context of aging or in response to anthracycline treatment [11,14,15]. Aging is associated with modifications of the cardiac stromal microenvironment characterized by senescence of cardiac mesenchymal stromal cells (cMSCs) sustained by inflammatory monocyte-derived CCR2^+^ macrophages and IL-1β production [16]. 

In this study, we show that cardiomyocyte-dependent oxidative stress induces premature senescence of cMSCs, and promotes the increase of non-senescent CCR2^+^ cardiac macrophages and of pro-inflammatory cytokine expression within the cardiac micro-environment, partially mimicking age-related changes. The oxidative stress-induced senescence pathways could interact with physiological aging and contribute to the increased susceptibility of the elderly to HF.

## 2. Results

### 2.1. MAOA Dependent Oxidative Stress in Cardiomyocytes Triggers Stress-Induced Premature Senescence in Cardiac Stromal Cells

**Figure 1:**
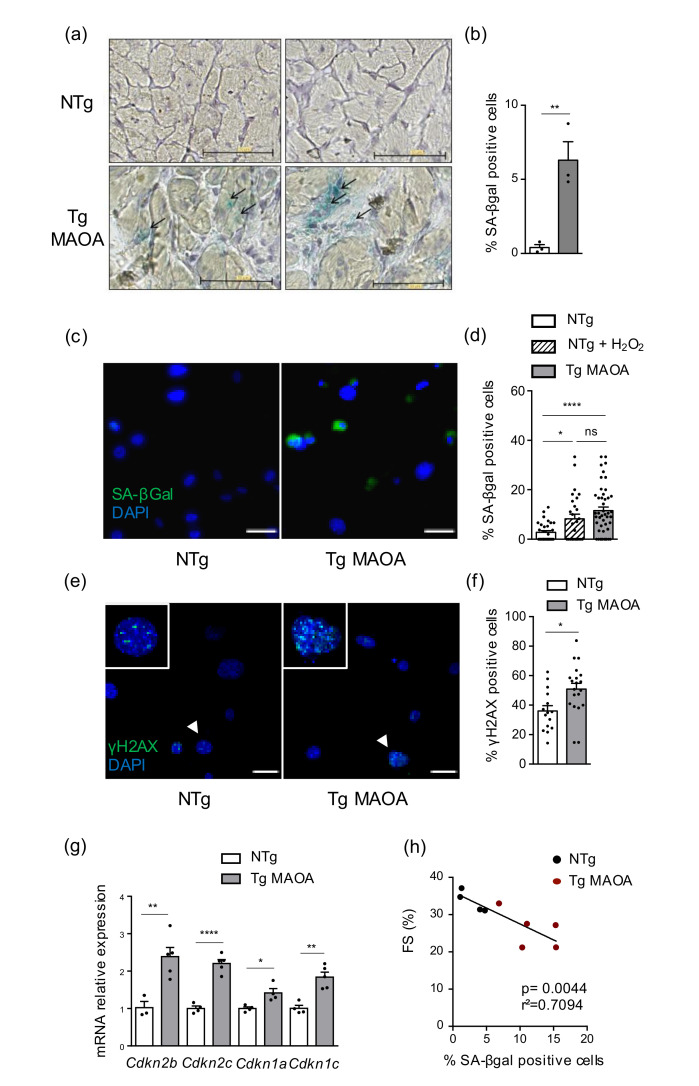
MAOA dependent oxidative stress in cardiomyocytes triggers stress induced premature senescence in cardiac stromal cells. (**a**,**b**) Representative images of SA-β Gal activity on heart cryosections of NTg and Tg MAOA mice (**a**) and quantification of the percentage of positive SA-β Gal cells per nuclei per field (**b**) (*n* = 3 mice per group). Scale bar, 50 µM. (**c**,**d**) Representative SA-β Gal staining (**c**, green) and percentage (**d**) of positive cardiac stromal cells from NTg (*n* = 8), NTg treated with 150µm H_2_O_2_ (*n* = 6) and Tg MAOA (*n* = 10) mice. DNA stained with DAPI (blue). Scale bar, 50 μm. (**d**,**e**) Representative immunostaining with anti-γH2AX antibody (**e**, green) and percentage (**f**) of positive cardiac stromal cells from NTg (*n* = 6) and Tg MAOA (*n* = 8) mice. DNA stained with DAPI (blue). White arrows indicated cells zoomed in. Scale bar, 50 μm. (**g**) Relative mRNA expression of CDKIs of cardiac stromal cells from Tg MAOA (*n* = 5) compared to NTg (*n* = 4) mice. (**h**) Correlation curve between percentage of positive SA-βGal cardiac stromal cells in vitro and fractional shortening (FS) of NTg (*n* = 4) and Tg MAOA (*n* = 5) mice. Data are expressed as means ± SEM. * *p* < 0.05, ** *p* < 0.01, **** *p* < 0.0001 vs. NTg group.

Excessive oxidative stress generated by MAOA activity overexpressed by cardiomyocytes of Tg MAOA mice promotes the installation of systolic dysfunction at three to four-month ages (Supplementary Table S1) [9]. To assess whether overexpression of MAOA in cardiomyocytes could promote SIPS of cardiac stromal cells in vivo, senescence-associated beta-galactosidase (SA-β Gal) activity was tested on heart cryosections. In Tg MAOA mice, some diffuse staining was evidenced in cardiomyocytes, albeit a predominant strong staining appeared in the non-cardiomyocyte stromal cell fraction, compared to Ntg mice (Figure 1a,b). These results were confirmed using primary cardiac stromal cell cultures that showed higher percentages of cells positive for SA-β Gal in Tg MAOA mice at the onset of cardiac dysfunction compared to Ntg mice (Figure 1c,d). In parallel, H_2_O_2_ treatment of NTg cardiac stromal cell cultures also increased SA-β Gal activity, showing that cardiac stromal cells are susceptible to undergo oxidative stress-induced premature senescence (Figure 1d). 

Senescence of cardiac stromal cells from Tg MAOA was confirmed by increased percentages of cells with DNA damage foci (γH2AX, Figure 1e,f) and by the upregulation of several CDKI gene expression from the INK4A family (*Cdkn2b* and *Cdkn2c*) and the CIP/KIP family (*Cdkn1a* and *Cdkn1c*) compared to Ntg mice (Figure 1g). Furthermore, we observed a strong positive correlation between the percentage of cardiac stromal cells positive for SA-β Gal and the reduction of fractional shortening (Figure 1g), supporting the hypothesis that senescence induction within the cardiac microenvironment is strongly associated with cardiac dysfunction.

These results demonstrated that oxidative stress in cardiomyocytes induced bystander senescence of cardiac stromal cells, which correlated to progressive cardiac dysfunction. 

### 2.2. SIPS of Cardiac Stromal Cells in Tg MAOA Mice Increased Expression of SASP Factors and Is Associated to Modification of Stromal Cell Subsets

**Figure 2:**
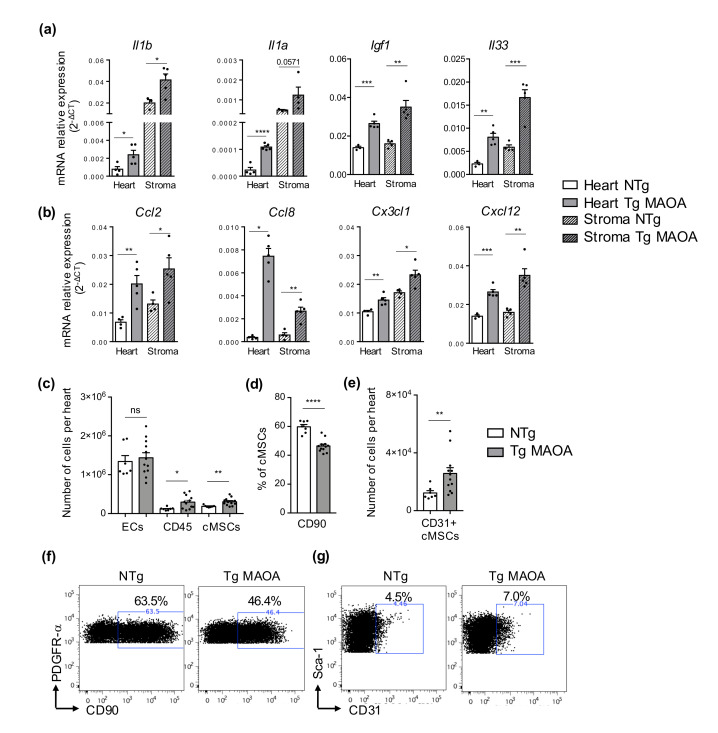
Premature senescence of cardiac stromal cells is associated to expression of SASP factors. (**a**,**b**) mRNA expression (2e^(−ΔCT)^) relative to gapdh of cytokines and growth factors (**a**) and chemokines (b) of heart and stromal cells from Tg MAOA (*n* = 5) and NTg (*n* = 4). (**c**) Numbers of endothelial cells (ECs), immune cells (CD45) and cardiac mesenchymal stromal cells (cMSCs) per heart ventricles of NTg (*n* = 7) and Tg MAOA (*n* = 12) mice analyzed by flow cytometry. (**d**) Percentage of CD90 positive cMSCs of NTg (*n* = 7) and Tg MAOA (*n* = 12) mice determined by flow cytometry. (**e**) Numbers of CD31^+^ cMSCs per heart ventricles of NTg (*n* = 7) and Tg MAOA (*n* = 12) mice by flow cytometry. (**f**,**g**) Representative dot-plots of PDGFR-α and CD90 (**f**) and of Sca-1 and CD31 (**g**) expression by cMSCs. Percentages of cells positive for CD90 (**f**) or for CD31 (**g**) out of total cMSCs from NTg and Tg MAOA mice are shown. Data are expressed as means ± SEM. * *p* < 0.05, ** *p* < 0.01, *** *p* < 0.001, **** *p* < 0.0001 vs. NTg group.

We asked whether oxidative stress derived from cardiomyocytes induced the expression of several mediators related to SASP acquisition in stromal cells. Gene expression of interleukins (*Il1a*, *Il1b*, *Il33*), *Igf1* (Figure 2a), and of several chemokines (*Ccl2*, *Cx3cl1*, *Ccl8*, and *Cxcl12*) (Figure 2b) was increased both in the heart and the stromal cell fraction of Tg MAOA mice compared to NTg mice. Interestingly, gene expression of the IL-1 family (*Il1a*, *Il1b*), known to mediate paracrine senescence [16], was higher in cardiac stromal cells compared to cardiac tissue of Tg MAOA mice, suggesting that non-cardiomyocyte cells were the major source of these pro-inflammatory cytokines (Figure 2a).

To determine how cardiomyocyte-dependent oxidative stress impacts the cardiac microenvironment, cardiac stromal cell populations of Tg MAOA mice were analyzed by flow cytometry. Whereas endothelial cell numbers were not significantly modified, Tg MAOA mice had increased numbers of cardiac immune cells (CD45+) and mesenchymal stromal cells (cMSCs) compared to NTg mice (Figure 2c). As for physiological cardiac aging [16,17], cMSCs of TgMAOA mice were modified compared to NTg, with decreased expression of CD90 (Figure 2d,f) and increased numbers of the rare hybrid cell population CD31^+^ cMSCs (Figure 2e,g).

These results showed that stress-induced premature senescence of cardiac stromal cells of Tg MAOA mice was associated with some of the features of physiological cardiac aging with modification of cMSC subsets and immune cells and with upregulation of pro-inflammatory gene expression.

### 2.3. Oxidative Stress-Induced Premature Senescence of Cardiac Mesenchymal Stromal Cells Shares Some Common Features with Physiological Aging-Associated Senescence 

**Figure 3:**
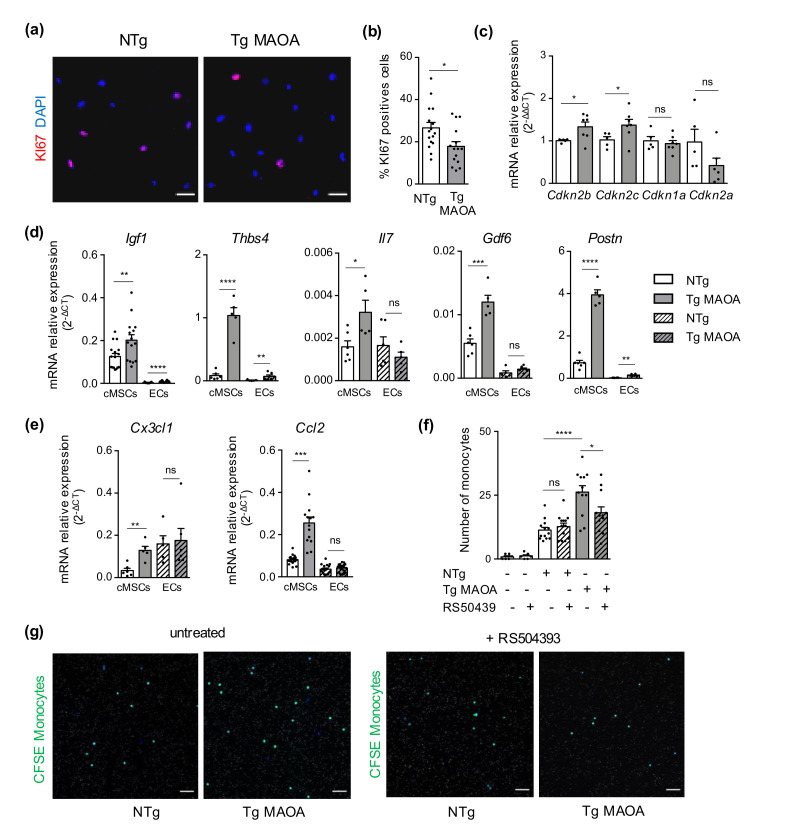
Oxidative stress induced premature senescence of cMSCs promotes CCR2-dependent monocyte recruitment. (**a**,**b**) Representative immunostaining of anti-Ki67 antibody (red; **a**) and percentage (**b**) of positive cMSCs isolated from NTg (*n* = 3) and Tg MAOA (*n* = 4) mice. DNA stained with DAPI (blue). Scale bar, 50 μm. (**c**) Relative mRNA expression of CDKIs from Tg MAOA cMSCs (*n* = 7) compared to NTg (*n* = 5). (**d**,**e**) mRNA expression of SASP factors (**d**) and of *Ccl2* and *Cx3cr1* chimiokines (**e**) by cMSCs and endothelial cells (ECs) isolated from NTg (*n* = 6) and Tg MAOA (*n* = 5) mice relative to gapdh gene expression. (**f**,**g**) Quantification and representative images of CFSE labelled monocytes pretreated or not with the CCR2 antagonist (RS 504393) at the lower part of the Fluoroblok insert after 2 h incubation with cMSCs from NTg (*n* = 3) or Tg MAOA mice (*n* = 4) in the bottom chamber compared to medium; nuclei: blue). Monocytes were pretreated (or not)). Nuclei were stained with DAPI (Blue), CFSE (green), DAPI (blue), pores: white. Scale bar, 50 μm. Data are expressed as means ± SEM. * *p* < 0.05, ** *p* < 0.01, *** *p* < 0.001, **** *p* < 0.0001 vs. NTg group.

We determined whether the modifications of cMSC population of Tg MAOA mice were associated with the induction of SIPS. cMSCs isolated from Tg MAOA mice showed a reduced proliferation rate (% Ki67; Figure 3a,b) and upregulated the expression of CDKI genes such as *Cdkn2b* and *Cdkn2c* (Figure 3c) compared to cMSCs of NTg mice. Concomitantly, cMSCs of Tg MAOA mice but not ECs expressed higher mRNA of several SASP mediators, previously identified during cMSC physiological aging, such as *Igf1*, *Thbs4*, *Il7*, *Gdf6*, and *Postn*, compared to Ntg mice (Figure 3d). Gene expression of *Ccl2* and *Cx3cl1* chemokines was also increased in cMSCs of Tg MAOA mice with specific induction of *Ccl2* expression in cMSCs compared to cardiac endothelial cells (EC) (Figure 3e). In conclusion, SASP acquisition by cMSCs of Tg MAOA mice, although with high inter-individual variability, was associated with decreased proliferation and induction of CDKI gene expression (*Cdkn2b* and *Cdkn2c*) strongly suggesting the triggering of SIPS by cMSCs.

We and others have previously shown that physiological aging induces modifications in the composition of cardiac stromal cells. In particular, senescence of cMSCs during aging favors the recruitment of monocytes by CCR2 activation and is associated with increased frequencies of CCR2^+^ cardiac macrophages and IL-1β production in the aging heart.

We then evaluated if SIPS of cMSCs had a functional impact on monocyte recruitment. Using a chemotaxis assay, we observed that cMSCs from Tg MAOA mice attracted more monocytes than cMSCs from NTg mice, and that inhibition of CCR2 by RS504393 prevented this gain of function (Figure 3f,g). These results showed that SIPS of cMSCs promoted monocyte recruitment through CCR2 activation, supporting a key role of senescent cMSCs in cardiac monocyte recruitment, comparable to physiological aging. 

### 2.4. Oxidative Stress-Derived Cardiomyocytes and SIPS of Cardiac Stromal Cells Promote CCR2^+^ Cardiac Macrophages and Expression of Cytokines of the IL-1 Family 

**Figure 4:**
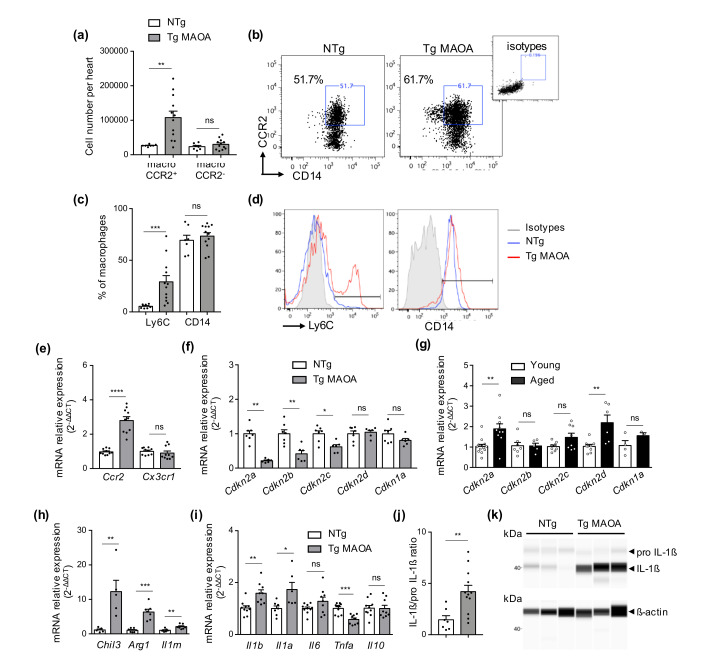
Cardiomyocyte oxidative stress triggers shift in macrophage population phenotype toward a mixed M1/M2 profile. (**a**) Numbers of CCR2^+^ and CCR2^-^ macrophages in ventricles of NTg (*n* = 7) and Tg MAOA mice (*n* = 12). (**b**) Representative dot-plots of CCR2 and CD14 expression by cardiac macrophages of NTg and Tg MAOA mice determined by flow cytometry. Control staining of viable macrophages with isotypes is shown. (**c**,**d**) Percentage of cardiac macrophages positive for Ly6C and CD14 of NTg (*n* = 7) and Tg MAOA (*n* = 12) mice (**c**) and representative histograms (**d**) by flow cytometry. (**e**) Relative mRNA expression of chemokine receptors, Ccr2 and Cx3cr1 by cardiac macrophages isolated from Tg MAOA (*n* = 10) compared to NTg (*n* = 10) mice. (**f**,**g**) Relative mRNA expression of CDKIs by cardiac macrophages isolated from Tg MAOA (*n* = 7) compared to NTg (*n* = 6) mice (**f**) or from 20 month-old mice (*n* = 7*–*9) compared to young (*n* = 5*–*9) mice (**g**). (**h**,**i**) Relative mRNA expression of M2 markers (*n* = 5*–*7 mice per group) (**h**) and of M1/M2 cytokines (*n* = 9*–*10 mice per group) (**i**) by cardiac macrophages isolated from Tg MAOA compared to NTg mice. (**j**,**k**) Quantification (**j**) of protein expression of IL-1β relative to pro IL-1*β* by cardiac macrophages isolated from NTg (*n* = 7) and Tg MAOA (*n* = 10) mice and representative images (**k**) of cell lysate samples immunoblotted with anti IL-1β (upper panel) or with anti *β*-actin (lower panel) by capillary-based western blot (*n* = 3 mice per group). Data are expressed as means ± SEM. * *p* < 0.05, ** *p* < 0.01, *** *p* < 0.001, **** *p* < 0.0001 vs. NTg groupe or (**f**) vs. young group.

Cardiac macrophages are known to play critical roles in tissue healing, adverse cardiac remodeling, and fibrosis. The impact of cardiomyocyte-specific oxidative stress on cardiac macrophage populations was evaluated by flow cytometry. Higher numbers of CCR2^+^ macrophages were observed in the hearts of Tg MAOA mice whereas CCR2^-^ macrophage numbers were unchanged (Figure 4a,b). Macrophages of Tg MAOA mice expressed CD14 and a higher percentage of Ly6C compare to Ntg mice (Figure 4c,d) concordant with the increased proportion of monocyte-derived CCR2^+^ macrophages in the cardiac microenvironment of Tg MAOA mice.

Analysis of cell-sorted cardiac macrophages confirmed higher Ccr2 gene expression compared to NTg mice while *Cx3cr1* gene expression was unchanged (Figure 4e). Conversely, CDKI gene expression of Tg MAOA macrophages was unchanged (*Cdkn1a*) or repressed (*Cdkn2a*, *Cdkn2b*, and *Cdkn2c*; Figure 4f) supporting that these macrophages did not undergo SIPS. Repression of CDKI expression was in sharp contrast with what was observed in physiological aging, as cardiac macrophages from aged mice (20-month-old) had significant induction of *Cdkn2a* and *Cdkn2b* gene expression compared to young (three-month-old) (Figure 4g). As for aging, increased frequency of CCR2^+^ macrophages in cardiac stroma of Tg MAOA mice was associated with the upregulation of gene expression of M2 markers (*Chil3*, *Arg1*, *Il1rn*; Figure 4h) and of M1 pro-inflammatory cytokines of the IL-1 family (*Il1a* and *Il1b*) but not of *Tnfa* compared to NTg mice (Figure 4i–k). These results showed that the cardiac microenvironment of Tg MAOA mice promoted cardiac macrophage shift toward CCR2^+^ monocyte-derived macrophages resulting of mixed M2 and M1 marker gene expression profile, as observed during aging [16], but without induction of macrophage senescence.

### 2.5. Macrophages Repressed Expression of Phagocytic Receptors in the Cardiac Microenvironment of Tg MAOA Mice 

**Figure 5:**
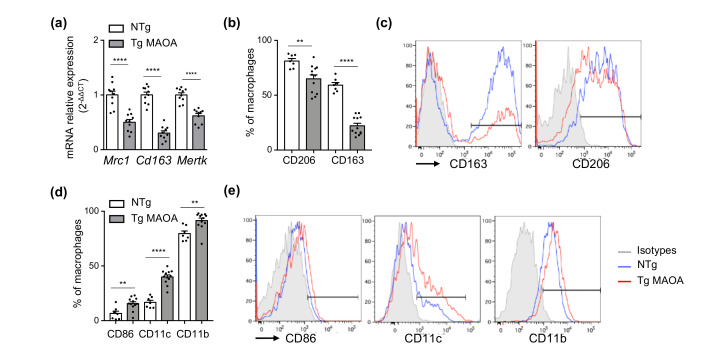
Cardiac macrophages of Tg MAOA mice have decreased expression of phagocytic receptors. (**a**) Relative mRNA expression of phagocytic receptors by cardiac macrophages of Tg MAOA compared to NTg mice (*n* = 10 mice per group). (**b**–**e**) Flow cytometry analysis of cardiac macrophages from NTg (*n* = 7) and Tg MAOA (*n* = 12) mice for M2 (**b**,**c**) and M1 (**d**,**e**) marker expression. Percentages of positive cells (**b**,**d**) and representative histograms (**c**,**e**) are shown. (**e**) Data are expressed as means ± SEM. ** *p* < 0.01, **** *p* < 0.0001 vs. NTg group.

As macrophages play a prominent role in the clearance of senescent cells, which is compromised during aging, we asked whether the accumulation of senescent cMSCs in the cardiac microenvironment of Tg MAOA could be related to decreased expression of phagocytic receptors by cardiac macrophages. Indeed, gene expression of different classes of phagocytic receptors, *Mrc1*, *Cd163*, and *Mertk* was significantly repressed by cardiac macrophages of Tg MAOA compared to NTg mice (Figure 5a). Flow cytometry analysis of cardiac macrophages confirmed the down-modulation of CD206 and CD163 expression in Tg MAOA mice, with decreased percentages of macrophages expressing high levels of these scavenger receptors associated with the M2-like phenotype (Figure 5b,c). Conversely, they up-regulated the expression of the inducible costimulatory molecule CD86 and the alpha chain integrins CD11c and CD11b, classically associated with M1-like phenotype, compared to macrophages of NTg mice (Figure 5d,e). However, most of the cardiac macrophages positive for CD86 also co-expressed CD206, strongly supporting the emergence of a macrophage subpopulation with a mixed M1/M2-like profile in Tg MAOA mice with decreased phagocytic functions.

## 3. Discussion

In this study, we demonstrated that, in the context of heart failure induced by oxidative stress, a senescence program was initiated in the heart and cardiac stromal cells. This stress-induced senescence program had similar features with senescence observed in cardiac stromal cells during physiological aging, with the accumulation of SA-β Gal positive cells, increased DNA damage (γH2AX) CDKI gene expression, and acquisition of SASP. Even if this program phenocopies most of the features of cardiac stromal cell aging, one striking difference is the lack of *Cdkn2a* induction, characteristic of late senescence and physiological aging. The difference could reside in the acute oxidative stress generated by cardiomyocyte MAOA overexpression in Tg MAOA mice, whereas a progressive increase of MAOA expression by cardiomyocytes during aging is expected to generate milder oxidative stress for longer periods.

Interestingly, the percentage of SA-β Gal in cardiac stromal cells was positively correlated with the severity of cardiac systolic dysfunction, showing that cardiac stromal cell senescence is associated with the progression of cardiac dysfunction. The factors related to SASP acquisition in the heart and stromal cells of Tg MAOA mice were enriched in pro-inflammatory mediators, such as cytokines of the IL-1 family (*Il1a*, *Il1b*), and monocyte chemo-attractant chemokines (*Ccl2*, *Ccl8*, and *Cx3cl1*). These factors participate in the installation of a chronic inflammatory micro-environment which is known to promote cardiovascular dysfunction [18,19]. Indeed, elevated serum pro-inflammatory cytokines and increased frequencies of blood intermediate monocytes have been shown to be predictive of worsened clinical outcomes in patients with heart failure in numerous studies [20,21].

In this study, we demonstrated that during cardiac dysfunction, cardiac mesenchymal stromal cells (cMSCs) expressed a stress-induced premature senescence program with decreased proliferative potential, induction of CDKI expression (*Cdkn2b*, *Cdkn2c*) but not of *Cdkn2a*, and of some SASP components previously identified in the context of cardiac aging (*Gdf6*, *Ccl2*, *Cx3cl1*, *Thbs4*, *Postn, IL7*) [16]. 

Due to this SASP production, cMSCs from Tg MAOA mice had greater potential to recruit monocytes by CCR2-dependent activation. CCR2^+^ monocytes are known to differentiate into pro-inflammatory monocyte-derived CCR2^+^ macrophages in the heart which in turn exacerbate this deleterious pro-inflammatory micro-environment [18,22]. These data suggested that oxidative stress-induced cardiac dysfunction and aging could share some common molecular pathways including senescence of stromal cells and a proinflammatory micro-environment. 

Cell sorted cardiac macrophages from Tg MAOA mice, expressed higher *Ccr2* mRNA levels than macrophages from NTg mice in accordance with the increased frequencies of CCR2^+^ macrophages in the CD45 population of Tg MAOA mice compared to NTg mice. Strikingly, even if this sub-population shift has been previously observed during physiological aging in the heart [16], the kidney [23], and other solid organs [24], expression of CDKI genes by cell sorted macrophages from Tg MAOA mice was unchanged or even reduced for *Cdkn2a* compared to control NTg mice. These results suggested that changes in CCR2^+^ macrophage frequencies were independent from the induction of an intrinsic senescence program but rather were the consequences of stress-induced senescence of cardiac stromal cells, in particular of cMSCs. Analysis of activation and differentiation markers of the cardiac macrophage pool from Tg MAOA mice revealed higher expression of classical M2 markers *Chil3*, *Arg1,* and *Il1rn* and lower expression of *Tnfa.* Despite this alternative activation profile, macrophages from Tg MAOA mice had decreased expression of phagocytic receptors (Mertk, CD206, and CD163) and increased expression of *Il1b* and of M1 surface markers such as CD11c, CD86, and CD11b. Phagocytosis of cardiomyocyte-derived fragments containing dysfunctional mitochondria by cardiac macrophages plays a key regulatory role in cardiac homeostasis and function by preserving cardiomyocyte fitness [25]. Mertk deficiency impairs the clearance of cardiomyocyte-derived damaged mitochondria by resident macrophages, promotes accumulation of free mitochondria in the extracellular space, and of dysfunctional mitochondria in cardiomyocytes, associated with alteration of cardiac function [25]. In Tg MAOA mice, the decreased expression of phagocytic receptors such as Mertk by cardiac macrophages could decrease the clearance of damaged mitochondria, which accumulate in cardiomyocytes overexpressing MAOA [9], and could so contribute to the installation of cardiac dysfunction. Modification of the cardiac macrophage pool could also participate in senescence spreading by decreasing the clearance of senescent cells and by favoring cMSC senescence through IL-1β secretion [16], promoting SASP exacerbation and progression toward systolic dysfunction. Indeed, previous reports have causally linked the cardiac CCR2^+^ macrophages subset which expresses IL-1β and pathological myocardial remodeling in both mice and humans [18,19]. Activation of the NLRP3 inflammasome is one possible mechanism driving IL-1β activation and secretion by macrophages of Tg MAOA mice as this pathway has been identified as a major contributor to age-related inflammation and cardiac aging [26,27]. Indeed, NLRP3 links mitochondria dysfunction and inflammation through NLRP3 priming and activation by mitochondrial DNA and ROS production [28,29]. Cardiomyocytes from Tg MAOA mice have been previously shown to present oxidation of mitochondrial DNA and severe mitochondria dysfunction exacerbating ROS production [9] that could favor the release of oxidized mitochondrial DNA fragments contributing to NLRP3 activation in macrophages. Moreover, endogenous MAOB-dependent ROS production by bone-marrow-derived macrophages has been shown to regulate NLRP3 activation [30]. Pharmacological inhibition of MAOB reduced IL-1β secretion in response to LPS/ATP-dependent inflammasome activation whereas NF-kB-dependent TNF-α production was unaffected [30], suggesting that excessive mitochondrial ROS production by Tg MAOA cardiomyocytes could have a direct role in macrophage NLRP3 activation. The implication of the NLRP3 pathway in Tg MAOA mice to trigger IL-1β production by cardiac macrophages and sustain cardiac stromal cell senescence will require further investigations.

In conclusion, our results have revealed a strong interplay between oxidative stress-induced senescence pathways and the installation of systolic cardiac dysfunction which involves the triggering of SIPS. A better understanding of microenvironment-dependent mechanisms concurring to cardiac dysfunction onset will help to define new targets to counteract the impact of aging on cardiac dysfunction and the increased susceptibility of the elderly to HF.

## 4. Materials and Methods

### 4.1. Animals

MAOA transgenic mice (Tg MAOA) backcrossed on the C57BL6/J background, with cardiac-specific overexpression of MAO-A driven by the α-MHC promoter have been previously described [9]. Male three- to four-month-old mice, at the onset of systolic dysfunction, were used for all experiments, with age-matched non-transgenic (NTg) littermates used as controls. The aging study was realized using three-month-old (young) or 20-month-old (aged) male C57Bl/6JRj mice (Janvier Laboratories, Le Genest-Saint-Isle, France). All mice were maintained under specific and opportunistic pathogen-free conditions and handled according to procedures performed in accordance with the recommendations of the European Accreditation of Laboratory Animal Care (86/609/EEC) and guidelines established by the Ethics and Animal Safety Committee of INSERM Toulouse/ENVT (agreement number: C31555011, 30 November 2020; experimental protocol number: CEEA-122 2014-81, 1 February 2015).

### 4.2. Echocardiography

Animals were anesthetized with 2% isoflurane and examined with noninvasive echocardiography (echocardiograph Vevo2100 (VisualSonics, Amsterdam, The Netherlands)). Cardiac ventricular dimensions were measured on M-mode images at least five times for the number of animals indicated and reported in Supplementary Table S1. 

### 4.3. Preparation of Cardiac Stromal Cell Suspensions

Hearts were harvested from mice after intraventricular perfusion of 10 mL PBS, minced with scalpels, and digested with Liberase TM (Roche, Boulogne-Billancourt, France) diluted in RPMI1640 (GIBCO, Thermo Fisher Scientific, Illkirch, France) as described previously [31]. Briefly, digestion of tissue fragments was performed by two successive incubations for 10 min with an enzymatic solution at 37 °C under shaking and stopped by the addition of heat-inactivated fetal calf serum (HIFCS) (Sigma–Aldrich, Merck, Darmstadt, Germany). Single-cell suspensions were obtained by filtrations on 100 µm and 40 µm cell strainers (BD Falcon). Red blood cells were removed by hypotonic shock with ammonium chloride solution (0.83%). Cardiac stromal cells were washed and used for qPCR analysis, flow cytometry analysis, or cell sorting or directly plated in MEMα 10% FBS. After 24 h, non-adherent debris were removed, and adherent cells were cultured further. Cells were used at P1 or P2.

### 4.4. Protein Quantification by Capillary-Based Western Blot

Quantification of IL1-β/pro IL-1β protein ratio of cardiac macrophages was performed by capillary-based “Simple Western System” (WES, ProteinSimple, Bio-Techne, Noyal Châtillon sur Seiche, France) according to the manufacturer’s protocol using goat anti-mouse IL-1b/IL-1F2 (1/250, AF-401-NA, R&D systems, Bio-Techne) as described previously [16]. Proteins were extracted from 50,000 sorted cMPs and protein loading was checked by β-actin quantification using rabbit anti-mouse β-actin (1/250, 4970, Cell Signaling Technology, Ozyme, Saint-Cyr-L’Ecole, France). Separation and immunoprobing were performed automatically and the chemiluminescent signals were detected and analyzed by Compass software (ProteinSimple, Bio-Techne).

### 4.5. Cardiac Stromal Cell Staining for Flow Cytometry

Cardiac stromal cells were incubated for 15 min at 4 °C in blocking buffer (DPBS (SIGMA) with 4% HIFCS, 3% mouse serum (SIGMA), 3% rat serum (SIGMA), and 2 mM EDTA (Euromedex, Souffelweyersheim, France)) with 5 µg/mL anti CD16/CD32 (Biolegend, Ozyme) as previously described [31]. Cells were then incubated on ice with conjugated monoclonal antibodies and diluted at their optimal concentrations (Supplementary Table S2) in FACS buffer (DPBS with 4% HIFCS, 2 mM EDTA) for 30 min (FACS) or 45 min (cell sorting). Necrotic cells were excluded by staining with Live/Dead Aqua or Live Dead Yellow fluorescent reactive dyes (Life Technologies, Thermo Fisher Scientific).

Cardiac stromal cell subsets were isolated by high-speed sorting with the BD InfluxTM cell sorter (BD Biosciences, Allschwil, Switzerland) and cMSCs were cell sorted based on CD45^–^ CD31^–^ Sca-1^+^ CD140a^+^ expression, macrophages on CD45^+^ CD64^+^ MHCII^+^ and vascular ECs were cell sorted on CD45^–^ CD140a^–^ CD31^+^ Sca-1^+^ expression (Supplementary Table S2).

### 4.6. Cell Immunofluorescence Assays and Confocal Microscopy

cMSCs were seeded on eight-well culture chamber slides (Lab-Tek II, Nunc, Thermo Fisher Scientific) at 10,000 cells per well. Cells were fixed with PFA 4% (Sigma–Aldrich, Merck) in DPBS. Cells were then incubated for 30 min in blocking buffer (DPBS with 5% FCS, 5% BSA, and 0.2% Triton ×100) and stained overnight at 4 °C with primary antibodies (Supplementary Table S2) diluted in antibody buffer (DPBS with 2% FCS, 2% BSA, and 0.2% Triton ×100). After three washes in DPBS 0.02% Tween20 for 10 min, staining with secondary antibodies (Supplemental Table S2) was performed for 1 h in antibody buffer at RT. Nuclei were stained with DAPI (Invitrogen, Thermo Fisher Scientific) for 10 min and slides were mounted with fluoromount G (Invitrogen, Thermo Fisher Scientific). Images were acquired with a LSM 780 confocal microscope (Zeiss, Marly le Roi, France). Three to four images were acquired per well and one image represented one field of acquisition. Images were analyzed with Fiji Software. For Ki67 and γH2AX stainings, results are expressed as the percentage of positive cells per field of acquisition. 

### 4.7. SA-β Galactosidase Activity

Heart cryo-sections (10 µm) were fixed and stained with the Senescence β-galactosidase Staining kit (Cell Signaling Technology, Ozyme; 9860) according to the manufacturer’s protocol with the following modifications. The β-galactosidase staining solution was prepared at pH 6 and added to each slide for 24 h at 37 °C, washed, counterstained with hematoxylin, and digitized with a Hamamatsu NanoZoomer (Hamamatsu, Japan). 

cMSCs were seeded at 5000 cells/well in 96-well plates and cultured for two days in αMEM 10% HIFCS and treated or not with 150 µM of H_2_O_2_ (Sigma–Aldrich, Merck) in αMEM 10% HIFCS for two days. SA-β galactosidase activity was quantified by fluorescence microscopy (Axiovision, Zeiss) following the manufacturer’s protocol (Cell Biolabs, San Diego, CA, USA; Quantitative Cellular Senescence Assay; CBA-232) after DAPI labeling. Images were analyzed with Fiji Software. Results are expressed as the percentage of positive cells per nuclei. 

### 4.8. Chemotaxis Assays

Cell-sorted cMSCs from individual mice were plated in 24-well plates at 30,000 cells per well and cultured in αMEM 10% FCS for five days. Monocytes were isolated from murine bone marrow cells after flushing of the tibia and femurs, using the EasySep™ Mouse Monocyte Isolation Kit according to the manufacturer’s protocol (#19861; STEMCELL Technologies, Grenoble, France). Monocytes were labeled with CFSE (10 ng/mL, Invitrogen, Thermo Fisher Scientific) for 15 min and pre-incubated, or not, with CCR2 antagonist (10 µM, RS504393; Tocris, Bio-Techne) for 20 min. Monocytes (1.5 × 10^5^) were added to 3 µm pore size Fluoroblok^TM^ inserts (Corning, Avon, France) on top of either cMSCs or control medium in 24-well plates. After three hours in the incubator at 37 °C 5% CO_2_, Fluorobloks were fixed with PFA 4%, and monocyte nuclei were stained with DAPI. Only monocytes that have passed through the filter were quantified after imaging with the LSM 780 confocal microscope (Zeiss). Four images were acquired and quantified per well. 

### 4.9. RT-PCR

Total RNA was extracted using ReliaPrep™ RNA Miniprep Systems (Promega, Charbonnières-les-Bains, France) and quantified by spectrophotometry (ND-100 NanoDrop, Thermo Fisher Scientific). cDNA was synthesized with either the MultiScribe™ reverse transcriptase (High-Capacity cDNA Reverse Transcription Kit, Applied Biosystem, Thermo Fisher Scientific), or, for experiments with low amounts of RNA, with SuperScript™ VILO™ cDNA Synthesis Kit (Invitrogen, Thermo Fisher Scientific). PCR was performed for 40 cycles with SYBR green (Takara, Ozyme) on a Viia7 (Thermo Fisher Scientific). Primer sequences were designed or checked using Primer-BLAST [32] and are listed in Supplementary Table S3. *Rplp0* and *Gapdh* were used as reference genes for normalization. Results are expressed as relative gene expression compared to a housekeeping gene (2^−∆^CT) or compared to the control group (2^−∆∆CT^), calculated using the comparative cycle threshold (CT) method.

### 4.10. Statistics

Results are expressed as means ± SEM. The statistical significance between two groups was estimated using either the unpaired student’s two-tailed t-test or with the nonparametric Mann–Whitney U test, when indicated in the figure legend. Multi-group comparisons were performed using either one-way ANOVA with Bonferroni’s post-hoc test or, when indicated, with the Kruskal–Wallis test with Dunn’s post-hoc test, for samples with a non-gaussian distribution. Differences between groups were tested using GraphPad Prism software (version 7; GraphPad, San Diego, CA, USA) and considered statistically significant for *p* < 0.05.

## Data Availability

The data presented in this study are available on request from the corresponding author.

## Supplementary Materials

Supplementary Tables can be found at https://www.mdpi.com/1422-0067/22/5/2245/s1.

## Abbreviations

cMSC	Cardiac Mesenchymal Stromal Cells
MAOA	Mono Amine Oxidase
MP	Macrophage
NTg	Non Transgenic
Tg	Transgenic
