# Supplementary material for: Selective Cardiomyocyte Oxidative Stress Leads to Bystander Senescence of Cardiac Stromal Cells
Source: Int J Mol Sci. 2021 Feb 24;22(5):2245. doi: 10.3390/ijms22052245 (PMC7956294; doi:10.3390/ijms22052245)
Supplement: Supplementary file 1 [file ijms-22-02245-s001.pdf]

Supplementary Tables

Supplementary Table S1: Echocardiographic Measurements of NTg and Tg MAOA mice at 9 and 13 weeks.
Data are expressed as means ± SEM. *p < 0.05, **p < 0.01 vs NTg

**Age**	9 weeks		13 weeks	
**Genotype**	NTg	Tg MAOA	NTg	Tg MAOA
**Number of animals**	5	8	5	7
**FS (%)**	33,76 ± 1,61	31,66 ± 1,76	34,08 ± 1,22	26,75 ± 1,61 **
**LVEF (%)**	63,15 ± 2,25	60,07 ± 2,67	63,92 ± 1,8	52,56 ± 2,65 **
**LV mass (mg)**	130,3 ± 5,93	129,6 ± 6,87	113,5 ± 5,34	138,6 ± 6,81 *
**LIVDd (mm)**	3,998 ± 0,16	3,856 ± 0,18	3,564 ± 0,15	3,996 ± 0,11 *
**LIVDs (mm)**	2,650 ± 0,12	2,694 ± 0,15	2,344 ± 0,21	2,848 ± 0,14
**Heart rate (bpm)**	559,5 ± 15,10	538,4 ± 10,97	563,1 ± 11,59	537,5 ± 8,686

FS, Fractional shortening; LVEF, left ventricular ejection fraction ; LV mass, left ventricular mass ; LVIDs, diastolic left ventricular dimension; LVIDs, systolic left ventricular dimension

Supplementary Table S2: Antibody list

Primary antibodies (Immunofluorescence)		
Target	Supplier	ref
Immunofluorescence microscopy		
Ki67 (Clone SP6)	Cell Marque	275R-14
γH2A.X (Clone JBW301)	Merck-Millipore	05-636
Goat anti mouse AF488	Life Technologies	A-11029
Goat anti rabbit AF594	Life Technologies	A-11037

Other		
DAPI	Sigma	D9542

Flow cytometry antibodies			
Cell sorting Panel			
Target	Clone	Supplier	Ref
CD45 APC-Cy7	30-F11	Biolegend	103116
CD31 PerCP-Cy5.5	390	Biolegend	102420
Sca-1 BV711	D7	Biolegend	108131
CD140a APC	APA5	Biolegend	135908
CD64 PE	X54-5/7.1	Biolegend	139303
MHCII FITC	M5/114.15.2	Biolegend	107606
Aqua Dye		Life technologie	L34966

cardiac macrophages			
CD45 APC-Cy7	30-F11	Biolegend	103116
CD64 PE-Cy7	X54-5/7.1	Biolegend	139313
CCR2 PE	475301	R&D Systems	FAB5538P
Ly6C eFluor450	HK1.4	eBioscience	48-5932
MHCII FITC	M5/114.15.2	Biolegend	107606
CD14 PerCP-eFluor710	Sa2-8	eBioscience	46-0141-82
CD11c AF647	N418	Biolegend	117314
CD86 PerCP-Cy5.5	GL-1	Biolegend	105027
CD206 AF647	MR5D3	Bio-rad	MCA2235A647T
CD163 PE	TNKUPJ	eBioscience	12-1631-80
CD11b PB	M1/70	Biolegend	101224
Yellow Dye		Life Technologies	L34959

Cardiac mesenchymal stromal cells			
CD45 APC-Cy7	30-F11	Biolegend	103116
CD31 PerCP-Cy5.5	390	Biolegend	102420
Sca-1 FITC	D7	Biolegend	108131
CD140a APC	APA5	Biolegend	135908
CD90.2 BV421	53-2.1	Biolegend	140327
Yellow Dye		Life Technologies	L34959

Supplementary Table S3: Mouse primer list

Primers					
Arg1	S	AGAGCTGACAGCAACCCTGT	*Igf1*	S	GCTCTTCAGTTCGTGTGTGGAC
	AS	GGATCCAGAAGGTGATGGAA		AS	AGCCTGTGGGCTTGTTGAAGTA
Ccl2	S	AAACCTGGATCGGAACCAAAT	*Il10*	S	TTCAGCCAGGTGAAGACTTTCT
	AS	TACGGGTCAACTTCACATTCAAA		AS	GCTTGGCAACCCAAGTAACC
Ccl8	S	CACCTGAGTTAAGAGACAGCCAAAG	*Il1a*	S	TTGGTTAAATGACCTGCAACA
	AS	TGAGAAAACACGCAGCCCA		AS	GAGCGCTCACGAACAGTTG
Ccr2	S	CCTTGGGAATGAGTAACTGTGTGA	*Il1b*	S	GATCCACACTCTCCAGCTGCA
	AS	AATGACAGGATTAATGCAGCAGTGT		AS	CAACCAACAAGTGATATTCTCCATG
Cd163	S	CATCATGGCACAGGTCACCC	*Il1rn*	S	ACATGGCAAACAACACAGGA
	AS	CGCTGAATCTGTCGTCGCTT		AS	TAGCAAATGAGCCACAGACG
Cdkn1a	S	GCAGAATAAAAGGTGCCACAGGC	*Il33*	S	ATGGGAAGAAGCTGATGGTG
	AS	CCGAAGAGACAACGGCACACT		AS	CCGAGGACTTTTTGTGAAGG
Cdkn1c	S	CTACGCGCTATCACTGGGAAG	*Il6*	S	GAGGATACCACTCCCAACAGACC
	AS	ATCACCAATCAGCCAGCCTT		AS	AAGTGCATCATCGTTGTTCATACA
Cdkn2a	S	CCGAACTCTTTCGGTCGTACCC	*Il7*	S	ACCATGTTCCATGTTTCTTTTAGAT
	AS	CTGCTACGTGAACGTTGCCCA		AS	TGTGACAGGCAGCAGAACAA
Cdkn2b	S	AGATCCCAACGCCCTGAAC	*Mrc1*	S	ATGCCAAGTGGGAAAATCTG
	AS	CAGTTGGGTTCTGCTCCGT		AS	TGTAGCAGTGGCCTGCATAG
Cdkn2c	S	GGGGGACCTAGAGCAACTTAC	*Mertk*	S	AAGGTCCCCGTCTGTCCTAA
	AS	CTCCGGATTTCCAAGTTTCA		AS	GCGGGGAGGGGATTACTTTG
Cdkn2d	S	CCTGAACCGCTTTGGCAAGAC	*Postn*	S	GGACACCTCGTGGCAGTTTC
	AS	ATCTTGGACATTGGGGCTGGC		AS	CCGCCATCACCTCTGACCT
Chi3l3	S	AATGATTCCTGCTCCTGTGG	*Thbs4*	S	TTCAGATGTGACGCCTGTCC
	AS	ACTTTGATGGCCTCAACCTG		AS	CAACATCAGTGCACACCTGC
Cx3cl1	S	TGGCTTTGCTCATCCGCTATCAG	*Tnfa*	S	CCACCACGCTCTTCTGTCTAC
	AS	CGTCTGTGCTGTGTCGTCTCC		AS	AGGGTCTGGGCCATAGAACT
Cx3cr1	S	GCCTGTTATTTGGGCGACATT			
	AS	GCGAGGACCACCAACAGATTT			
Cxcl12	S	CTCAACACTCCAAACTGTGCC	*Rplp0*	S	GCTTCATTGTGGGAGCAGAC
	AS	TTGGGCTGTTGTGCTTACTTG		AS	ATGGTGTTCTTGCCCATCAG
Gdf6	S	GGGCATCAATGCCAGCTTTT	*Gapdh*	S	ATGACTCCACTCACGGCAAATT
	AS	GTGCGAGAGATCGTCCAGTC		AS	TCCCATTCTCGGCCTTGAC
